# Tracking sensory system atrophy and outcome prediction in spinal cord injury

**DOI:** 10.1002/ana.24508

**Published:** 2015-09-18

**Authors:** Patrick Grabher, Martina F. Callaghan, John Ashburner, Nikolaus Weiskopf, Alan J. Thompson, Armin Curt, Patrick Freund

**Affiliations:** ^1^ Spinal Cord Injury Center BalgristUniversity Hospital Zurich, University of ZurichZurichSwitzerland; ^2^ Wellcome Trust Centre for NeuroimagingUCL Institute of Neurology, University College LondonLondonUnited Kingdom; ^3^ Department of NeurophysicsMax Planck Institute for Human Cognitive, and Brain SciencesLeipzigGermany; ^4^ Department of Brain Repair and RehabilitationUCL Institute of Neurology, University College LondonLondonUnited Kingdom

## Abstract

**Objective:**

In patients with subacute spinal cord injury (SCI), the motor system undergoes progressive structural changes rostral to the lesion, which are associated with motor outcome. The extent to which the sensory system is affected and how this relates to sensory outcome are uncertain.

**Methods:**

Changes in the sensory system were prospectively followed by applying a comprehensive magnetic resonance imaging (MRI) protocol to 14 patients with subacute traumatic SCI at baseline, 2 months, 6 months, and 12 months after injury, combined with a full neurological examination and comprehensive pain assessment. Eighteen controls underwent the same MRI protocol. T1‐weighted volumes, myelin‐sensitive magnetization transfer saturation (MT), and longitudinal relaxation rate (R1) mapping provided data on spinal cord and brain morphometry and microstructure. Regression analysis assessed the relationship between MRI readouts and sensory outcomes.

**Results:**

At 12 months from baseline, sensory scores were unchanged and below‐level neuropathic pain became prominent. Compared with controls, patients showed progressive degenerative changes in cervical cord and brain morphometry across the sensory system. At 12 months, MT and R1 were reduced in areas of structural decline. Sensory scores at 12 months correlated with rate of change in cord area and brain volume and decreased MT in the spinal cord at 12 months.

**Interpretation:**

This study has demonstrated progressive atrophic and microstructural changes across the sensory system with a close relation to sensory outcome. Structural MRI protocols remote from the site of lesion provide new insights into neuronal degeneration underpinning sensory disturbance and have potential as responsive biomarkers of rehabilitation and treatment interventions. Ann Neurol 2015;78:Ann Neurol 2015;78:679–696

Traumatic spinal cord injury (SCI) leads in most incidences to instantaneous loss of sensory input below the level of injury and permanent paralysis.^1^ No effective treatments are currently available, although limited motor and sensory recovery can be promoted by intensive rehabilitation, with the greatest improvements occurring during the first year after injury.^2^ During this time, disabling sensory discomfort and neuropathic pain below the level of lesion frequently develops as a secondary complication in SCI patients, severely impacting on patients' quality of life and functional independence.^3^, ^4^ The underlying mechanisms influencing sensory impairment and its outcomes are thought to relate to structural changes including axonal degeneration and demyelination,^5^ transneuronal atrophy,^6^ but also rewiring^7^ and hyperexcitability of neuronal circuits.^8^

Within the descending motor system, progressive structural changes have been directly linked to the recovery of muscle strength and functional independence during the first year following SCI.^9^ However, the structural correlates and time course of changes in sensory impairment and emergence of neuropathic pain within the injured spinal cord, as well as the brain, are less well defined. Cross‐sectional studies in chronic SCI have shown that sensory impairment and neuropathic pain below the level of the lesion correlated with structural^10^, ^11^, ^12^, ^13^, ^14^, ^15^ and functional changes^16^, ^17^ within the sensory system. However, such cross‐sectional studies in chronic SCI do not allow for assessment of the spontaneous evolution of structural and functional changes attributable to (1) the acute onset of deafferentation,^18^, ^19^, ^20^ (2) spontaneous partial sensory recovery, or (3) relearning of compensatory approaches relevant for activities of daily living (ie, visual inputs).^21^ Therefore, there is only limited knowledge about the temporal dynamics and specificity of trauma‐induced structural changes and their link to the arising sensory impairment and outcome and below‐level neuropathic pain within the spinal cord, brainstem, and brain.^22^

Recent advances in quantitative neuroimaging of the spinal cord and brain provide the possibility of monitoring temporal changes of the macrostructure as well as the microstructure from the earliest onset of SCI.^23^, ^24^ In a longitudinal prospective design, we used advanced magnetic resonance imaging (MRI) outcome measures to assess the spontaneous time course of structural progressive changes within the sensory system above the spinal level of the lesion (ie, cervical cord and brain). We measured next to cross‐sectional spinal cord area^9^ the anterior–posterior width (APW) and left–right width (LRW) at the identical cord level to provide detailed insights into morphometric cord changes.^12^ At the level of the brain, we applied tensor‐based morphometry to assess dynamic volumetric changes^25^ and voxel‐based quantification (VBQ) of magnetization transfer saturation (MT) and longitudinal relaxation rate (R1) maps^26^ to gain information about myelin integrity at 12‐month follow‐up. We hypothesized that specific macrostructural and microstructural changes appear in the sensory system early after traumatic SCI and that these changes would be associated with the extent of sensory impairment/outcome and the development of neuropathic pain below the level of injury.

## Subjects and Methods

### Subjects and Study Design

Fourteen patients with a subacute (<2 months postinjury) traumatic SCI (Table) and 18 healthy control subjects were recruited at the University Hospital Balgrist between July 2010 and June 2013. All patients fulfilled the following inclusion criteria: subacute (<2 months postinjury) traumatic SCI without head and brain lesions, no pre‐existing neurological, mental, or medical disorders affecting functional outcome, and no contradictions to MRI.

A comprehensive and detailed clinical protocol and pain questionnaire were performed on patients at baseline, 2 months, 6 months, and 12 months to assess their sensory and motor impairments. This protocol included the International Standards for the Neurological Classification of Spinal Cord Injury (ISNCSCI) protocol^27^ for motor, light touch, and pinprick score, and the Spinal Cord Independence Measure (SCIM).^28^ Using the European Multicenter Study about Spinal Cord Injury (EMSCI) pain questionnaire (v4.2, http://www.emsci.org/),^29^, ^30^ we assessed multiple aspects of pain (eg, onset, duration, maximal and average pain intensity, quality of pain [eg, nociceptive or neuropathic]) at each time point. To be classified as below‐level neuropathic pain, ongoing pain had to be located 3 or more segments below the level of lesion. Pain intensity was assessed using an 11‐point numeric rating scale from “0” indicating no pain to “10” indicating the worst pain imaginable pain.

All participants underwent a comprehensive MRI protocol at the same time points. The relationship between SCIM and ISNCSCI motor scores and structural changes have been reported previously in a subgroup of the present study cohort.^9^

Informed written consent was obtained from all participants before participation. The study protocol was in accordance with the Declaration of Helsinki and was approved by the local ethics committee of Zurich (EK‐2010‐0271).

**Table 1:** Clinical and Behavioral Data of 14 Patients with Subacute Traumatic Spinal Cord Injury

		Injury		ISNCSCI Grade at Baseline	Initial Site of Impairment, Motor/Sensory	ISNCSCI Pinprick		ISNCSCI Light Touch	
ID	Age at Injury, yr	Type	Severity			Baseline, Left/Right	12 Months, Left/Right	Baseline, Left/Right	12 Months, Left/Right
1	19	Fall	Complete	A	C5/C4	13/13	16/17	12/12	16/17
2	23	Fall	Incomplete	B	C7/C6	18/20	19/18	34/35	35/37
3	70	Fall	Incomplete	B	T10/T10	37/38	NA^a^	40/38	NA^a^
4	75	Fall	Incomplete	D	T12/T12	52/50	NA^b^	56/55	NA^b^
5	44	Fall	Incomplete	D	T11/T11	56/53	55/51	56/51	55/51
6	42	Fall	Complete	A	C5/C5	10/10	10/8	13/14	9/11
7	71	Fall	Incomplete	B	C7/C8	16/20	22/19	42/43	56/56
8	20	MVA	Complete	A	C5/C5	10/9	15/11	10/11	29/24
9	30	MVA	Incomplete	B	C7/C8	17/18	18/19	33/31	35/35
10	52	Fall	Incomplete	D	T9/T9	45/44	45/45	48/47	45/45
11	42	MVA	Incomplete	D	C5/C4	53/51	56/41	52/52	56/42
12	29	Fall	Complete	A	T11/T11	44/44	38/40	43/43	41/41
13	70	MVA	Complete	A	T7/T7	28/37	31/37	35/37	31/36
14	52	MVA	Incomplete	B	C6/C6	19/19	30/14	32/33	33/34

All patients were male, except Patient 3.

a No sensory testing was performed.

b Patient died.

ISNCSCI = International Standards for the Neurological Classification of Spinal Cord Injury. MVA = motor vehicle accident; NA = not available.

### Image Acquisition

Participants were scanned with a 3T Magnetom Verio MRI scanner (Siemens Healthcare, Erlangen, Germany) operating with a 16‐channel radiofrequency (RF) receive head and neck coil and RF body transmit coil. All participants were carefully positioned in the same position at all times by the radiographers to obtain high reproducibility across participants and time points to exclude any bias related to potential gradient nonlinearity over time at the level of the spinal cord.

T1‐weighted (T1w) structural whole‐brain volumes including the cervical cord to C5 were collected at each time point. At 12 months, we acquired additional data using a multiparameter mapping (MPM) quantitative MRI (qMRI) protocol.^26^ Data collected using this protocol can be used to compute maps of R1^31^ and MT.^32^ These metrics are physical MRI parameters, respectively describing the relaxation and magnetization transfer behavior of protons within their microenvironments. Both processes are enhanced by the presence of macromolecular content (eg, myelin). As such, they are sensitive to tissue microstructure and provide quantitative measures that can be used for multicenter studies and give information about myelin, water, and iron content.^33^ All image volumes were checked for artifacts.

For each subject, a 3‐dimensional (3D) T1w scan (magnetization‐prepared rapid acquisition gradient echo [MPRAGE])^34^ with 176 partitions was acquired at 1mm isotropic resolution in 9 minutes using the following parameters: field of view = 224 × 256 mm^2^, matrix size = 224 × 256, repetition time (TR) = 2,420 milliseconds, echo time (TE) = 4.18 milliseconds, inversion time = 960 milliseconds, flip angle α = 9°, and readout bandwidth = 150 Hz per pixel.

The quantitative MPM data were derived from 3 differently contrast‐weighted 3D multiecho fast low‐angle shot (FLASH) volumes acquired with 1mm isotropic resolution and a field of view of 240 × 256 mm^2^ (matrix size = 240 × 256) with 176 partitions in a total scan time of 23 minutes. To reduce the overall acquisition time, parallel imaging with a speed‐up factor of 2 was used in the phase‐encoding direction (anterior–posterior) using a generalized autocalibration partially parallel acquisition algorithm (GRAPPA). Additionally, a partial Fourier acquisition with a 6/8 sampling factor was used in the partition direction (left–right). Predominantly T1 weighting was achieved with TR = 25 milliseconds and α = 23°, whereas proton density (PD) weighting was achieved with TR = 25 milliseconds and α = 4°. Magnetization transfer weighting (TR = 37 milliseconds, α = 9°) was achieved by applying an off‐resonance RF pulse prior to nonselective excitation. The readout bandwidth was 480Hz per pixel. Seven equidistantly spaced echoes were acquired with TE ranging from 2.46 milliseconds to 17.22 milliseconds for all volumes. One additional echo was acquired at 19.68 milliseconds for both the PD‐weighted (PDw) and T1w volumes.

### Image Analysis

#### Changes to the Macrostructure and Microstructure of the Cervical Cord

We investigated remote trauma‐induced structural changes within the spinal cord at cervical level C2/C3. In addition to the previously reported cross‐sectional spinal cord area,^9^ we here assessed its change in the shape, which we parameterized with APW and LRW, because reductions in these are related to sensory and motor impairment, respectively.^12^

We used JIM 6.0 (Xynapse Systems, Aldwincle, UK) to extract 10 contiguous and reformatted axial slices of 3 mm at the C2/C3 level from the structural MPRAGE T1w volume. The cross‐sectional cord area was then calculated automatically with an active‐surface model.^35^ An ellipse was fit to the boundary of this spinal cord area, defined in the previous step in MATLAB (MathWorks, Natick, MA) to extract APW (elliptical short axis) and LRW (elliptical long axis).

To assess changes to the myelin content at the identical cervical cord level, we used in‐house MATLAB scripts based on nearest‐neighbor region growing to define the cord volume (stopping criterion: 40% drop in parameter value) within the MT map followed by the same ellipse‐fitting procedure. This region of interest (ROI) for the spinal cord was superimposed on the R1 maps and used to extract the mean quantitative parameters from the MT and R1 maps (processing of quantitative maps is explained in the next section).

#### Changes to the Macrostructure and Microstructure of the Brain

We used tensor‐based morphometry, implemented in SPM12 (Wellcome Trust Centre for Neuroimaging, University College London, London, UK), to investigate dynamic volumetric brain changes in patients and controls over time. This was performed with the MPRAGE T1w images obtained at baseline, 2 months, 6 months, and 12 months. For each participant, the 4 volumes were aligned longitudinally to their midpoint average using inverse‐consistent 3D nonlinear registration.^25^ This generated Jacobian determinant maps for each time point, as well as the participant's average image (in terms of both shape and intensity). Unified segmentation was used to segment the average image into gray matter, white matter, and cerebrospinal fluid.^36^ The Jacobian determinant maps were transformed to Montreal Neurological Institute (MNI) space using deformations derived from the fast diffeomorphic image registration algorithm (Dartel).^37^ The spatially normalized Jacobian maps were finally smoothed with an isotropic Gaussian kernel filter with 2mm full width at half maximum (FWHM). The processed data encoded volumetric expansion and compression in each participant.^9^

We used VBQ^26^, ^38^ to investigate the cross‐sectional differences in myelin integrity between patients and controls at 12 months. MT‐weighted, PDw, and T1w FLASH volumes were used to calculate quantitative parameter maps of MT and R1, which are sensitive to microstructural features of the tissue.^23^ Whereas MT maps are primarily sensitive to macromolecular content, most notably myelin,^32^, ^39^ R1 maps are additionally sensitive to free water content, axon diameter, and iron content.^31^, ^40^

UNICORT was used for bias estimation and correction of RF transmit field inhomogeneity effects in the R1 maps.^26^ The MT maps for each participant were segmented into gray matter, white matter, and cerebrospinal fluid using unified segmentation.^36^ The transformation to MNI space was performed using Dartel.^37^ Finally, the MT and R1 maps were warped to MNI space with the participant‐specific flow fields from the MT maps obtained with Dartel and smoothed with an isotropic Gaussian kernel filter with 3 mm FWHM. The VBQ approach was used for this normalization process to minimize partial volume effects.^38^

Because we were interested in trauma‐induced degeneration in the ascending sensory system, we defined specific ROIs. The subcortical and cortical ROIs were defined as a single ROI encompassing the bilateral anterior cingulate cortex (ACC), thalamus, primary and secondary (S2) somatosensory cortex, and insula to include the main sensory and pain modulatory structures,^15^, ^41^, ^42^, ^43^, ^44^ using the anatomy toolbox for SPM.^45^ The brainstem and cerebellum were defined as a further ROI using the SUIT toolbox for SPM.^46^ Regions were chosen according to whether they contain/receive ascending sensory pathways.^47^, ^48^, ^49^, ^50^

### Statistical Analysis

Stata 13 (StataCorp, College Station, TX) was used for statistical analysis of all nonbrain data. We estimated the rates of change of clinical impairments in patients with linear regression models, with time as predictor. To accommodate nonlinear recovery, time was modeled on a logarithmic scale. We assessed the rate of change of the spinal cord parameters using linear regressions in all participants. A group indicator, group × time, and time × time interaction were included in the regression model to compare the rates of change and to accommodate quadratic effects. Age, sex, and their interaction with time were used to diminish any confounding (linear) effects. Two‐sample *t* tests were used to assess MT and R1 differences between patients and controls in the spinal cord at 12 months.

Linear and non‐linear (quadratic) regression models in SPM12 were used to assess longitudinal brain volume changes in gray and white matter, and the microstructure at 12 months, in the defined ROIs. The analyses included a group indicator and time. To assess non‐linear changes, time was modelled as the quadratic term. Age was treated as a covariate of no interest. Statistical parametric maps were initially thresholded with an uncorrected voxel threshold of *p* = 0.001. To account for multiple testing, only clusters surpassing a corrected cluster threshold of *p* = 0.05 (familywise error corrected based on Gaussian random field theory) were considered as significant (unless otherwise stated for peak voxel)^51^ and reported in Results. One‐tailed *t* tests with a significance threshold of *p* < 0.05 were used in each voxel of interest to test for decreases in patients and to compare the integrity of myelin between controls and patients, using the 12‐month MT and R1 data. To ensure that each voxel was analyzed only once, either in the gray matter or white matter segments, explicit masks for each subspace were generated by assigning each voxel with a probability >20% to the tissue class with the highest probability.^52^ After characterizing the average group effects, we explored regional structural correlates of sensory function. We used the linear and quadratic coefficients derived from the aforementioned regression models as response variables and clinical outcomes at 12 months in patients as dependent variables. Age and lesion level were modeled as confounds. These associations were tested with F statistics. Only significant associations with p values of less than 0.05 are reported.

## Results

Fourteen patients with subacute traumatic SCI (13 men and 1 woman), with a mean age of 45.6 years (standard deviation [SD] = 20.0), and 18 healthy participants (12 men and 6 women), with a mean age of 34.1 years (SD = 9.5), were enrolled in this study (see Table). There was no statistically significant difference between the mean ages in the 2 groups (Mann–Whitney *U* test *p* = 0.138). Eight patients suffered from a tetraplegia (3 with a complete lesion) and 6 from paraplegia (2 with a complete lesion) according to the ISNCSCI classification.

The mean interval from the time of injury to the baseline scan was 45.93 days (SD = 18.38), to the second scan 96.64 days (38.09), to the third scan 209.46 days (59.14), and to the last scan 380.54 days (109.32). In total, 122 data sets were included, of which 32 were acquired at baseline, 29 at 2 months, 31 at 6 months, and 30 at 12 months. Thus, 95.3% of planned assessments were accomplished.

Besides the improvements in ISNCSCI motor score and SCIM score (as reported for this patient cohort earlier^9^), patients did not recover on the ISNCSCI pinprick (increase of 0.046 per log month, *p* = 0.967) and ISNCSCI light touch (increase of 1.439 per log month, *p* = 0.324) scores. Neuropathic pain below the lesion emerged in 6 patients, and their pain intensity increased over time on the EMSCI pain questionnaire (mean pain intensity increased by 0.71 per log month, 95% confidence interval [CI] = 0.072–1.341, *p* = 0.029).

### Changes to the Macrostructure and Microstructure of the Cervical Cord

**Figure 1:**
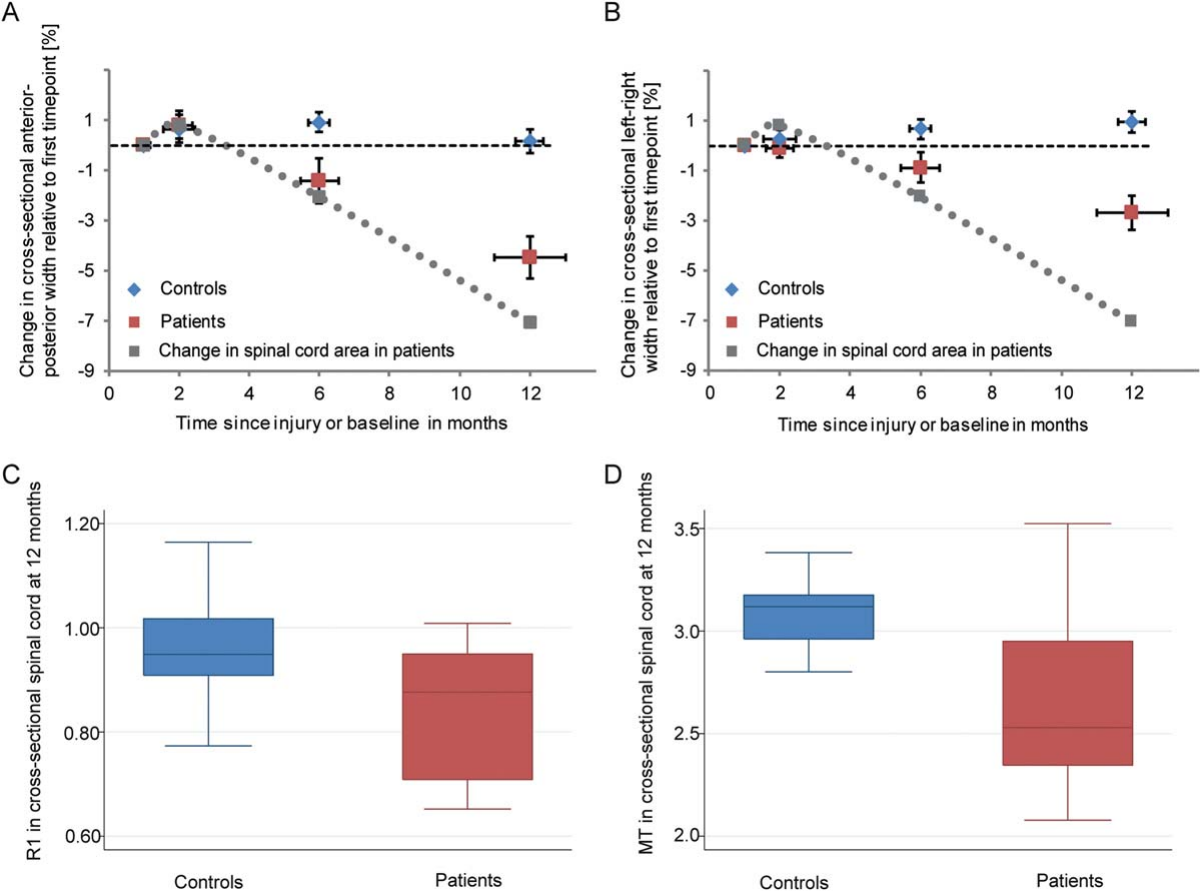
Longitudinal shape and cross‐sectional microstructural changes of the spinal cord above the lesion level at C2/C3 in patients (red) compared to controls (blue). (A) Shrinkage of the anterior–posterior width (APW) in patients compared to controls. (B) Shrinkage of the left–right width (LRW) in patients. No significant change was detected in controls. Vertical error bars show standard error (SE) for change in APW and LRW and horizontal error bars show SE for scan intervals. (C, D) Reduction of mean longitudinal relaxation rate (R1) and magnetization transfer saturation (MT) respectively at cervical C2/C3 level in patients compared to controls. Note that the results from the cross‐sectional cord area change has been reported previously^9^ and are shown only for illustrative purposes.

In addition to progressive decrease in overall cord area at C2/C3 above the lesion level in patients,^9^ we found spatially specific dynamic shape changes of the APW and LRW at the identical level (Fig 1A, B) between patients and controls (*p* < 0.001). In patients, the decrease of the APW was 0.022 mm per month (95% CI = −0.041 to −0.003, *p* = 0.023) and LRW decreased by 0.034 mm per month (95% CI = −0.059 to −0.010, *p* = 0.005). In controls, the cord metrics did not change over time (*p* = 0.238 for cord area, *p* = 0.136 for APW, *p* = 0.412 for LRW). In patients, the rate of decrease was similar between the APW and LRW (*p* = 0.520).

At the cervical cord level at 12 months, myelin‐sensitive MT and R1 were reduced in patients (MT: 2.65%, 95% CI = 2.40–2.90, *p* = 0.003; R1: 0.848 seconds^−1^, 95% CI = 0.769–0.926, *p* = 0.012) compared to controls (MT: 3.08%, 95% CI = 3.00–3.16; R1: 0.968 seconds^−1^, 95% CI = 0.916–1.020) by 14.96% and 12.41%, respectively (see Fig 1C, D).

### Changes to the Macrostructure and Microstructure in the Brain

**Figure 2:**
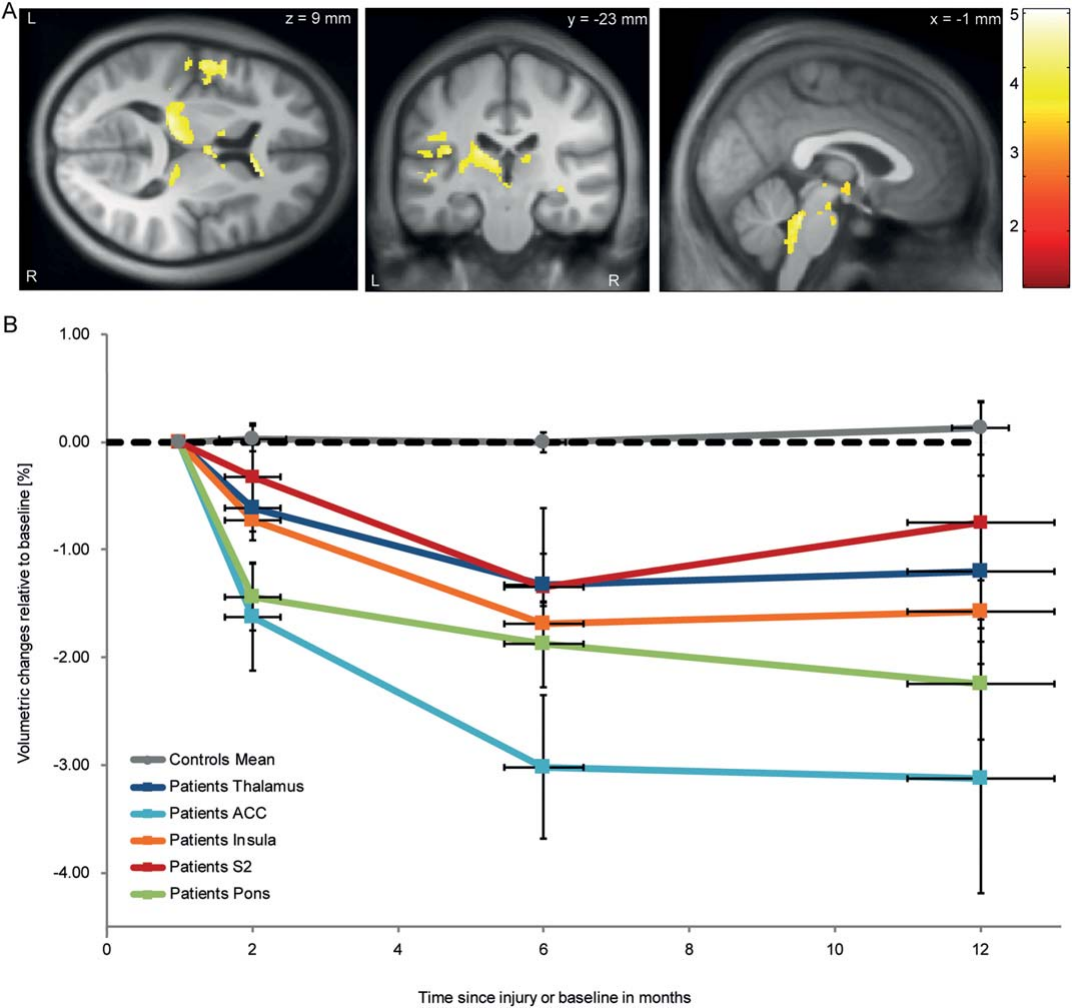
Longitudinal volumetric decreases in subcortical and brainstem gray and white matter shown by tensor‐based morphometry. (A) Overlay of statistical parametric maps (uncorrected *p* < 0.001, for illustrative purposes) showing volumetric decreases in gray and white matter. The color bar indicates the *t* score. (B) Illustration of progressive volumetric changes relative to baseline extracted from selected areas of interest. Vertical error bars show standard error (SE) for volumetric change in selected area of interest, and horizontal error bars show SE for scan intervals. ACC = anterior cingulate cortex; S2 = secondary somatosensory cortex.

Progressive focal brain volume decreases of up to 3% in the right thalamus (x: 20, y: −35, z: 2, *z* score = 4.20, *p* = 0.037, cluster extent = 287; x: 2, y: −5, z: 8, *z* score = 3.82, *p* = 0.019, cluster extent = 358), left thalamus (x: −20, y: −30, z: 9, *z* score = 3.98, *p* < 0.001, cluster extent = 1324), right ACC (x: 17, y: 30, z: 3, *z* score = 3.89, *p* = 0.003, cluster extent = 586), left ACC (x: −15, y: 28, z: −1, *z* score = 3.62, *p* = 0.010, cluster extent = 432), left insula (x: −38, y: −24, z: 17, *z* score = 3.85, *p* < 0.001, cluster extent = 1,028), left S2 (x: −54, y: 7, z: 5, *z* score = 4.09, *p* < 0.001, cluster extent = 842), and pons (x: 2, y: −39, z: −31, *z* score = 4.62, *p* < 0.001, cluster extent = 926) developed over time in patients compared to controls (Fig 2).

**Figure 3:**
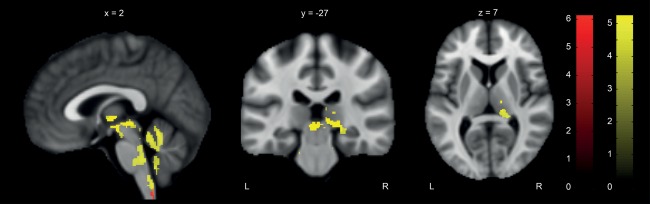
Changes in microstructure at 12 months revealed by voxel‐based quantification. Overlay of statistical parametric maps (uncorrected *p* < 0.001, for illustrative purposes) shows reduced magnetization transfer saturation (MT; red) and longitudinal relaxation rate (R1; yellow) in patients compared to controls at 12 months in thalamus, cerebellum with a cluster extending into the brainstem (ie, medulla oblongata, pons, and midbrain), and medulla oblongata (ie, dorsal column). These reductions suggest microstructural changes in patients, because MT and R1 are both sensitive to myelin. L = left; R = right.

At 12 months, myelin‐sensitive R1 was reduced in the thalamus by up to 19% (x: 0, y: −26, z: −3, *z* score = 4.10, *p* < 0.001, cluster extent = 959). R1 was also reduced by 20% in the left (x: 9, y: −45, z: −30, *z* score = 4.39, *p* < 0.001, cluster extent = 1,940) and by 17% in the right cerebellum (x: 2, y: −53, z: −14, *z* score = 4.31, *p* < 0.001, cluster extent = 3,124). The latter cluster extends into the brainstem (eg, medulla oblongata, pons, and midbrain). Myelin‐sensitive MT was reduced by 14% in the spinal cord dorsal columns (x: 0, y: −50, z: −66, *z* score = 4.80, *p* = 0.045, cluster extent = 51, only significant at peak voxel; Fig 3).

### Association between Structural Changes and Clinical Outcomes

**Figure 4:**
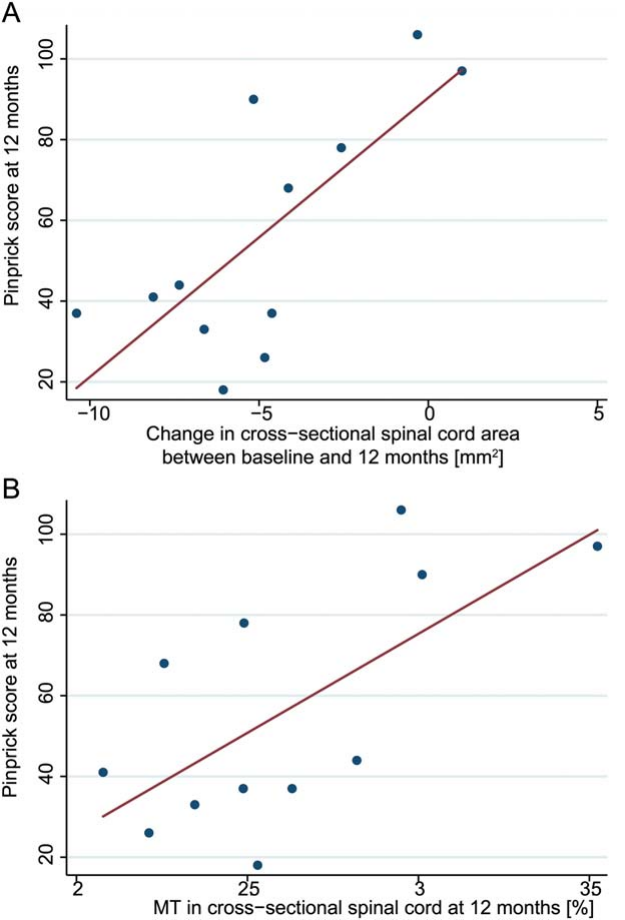
Correlations between structural changes in spinal cord and sensory outcome. Correlation is shown between pinprick score and change in cross‐sectional spinal cord area between baseline and 12 months (A) and magnetization transfer saturation (MT) at 12 months (B). Note, for illustrative purpose we used the unadjusted values of pin‐prick score and cord area change. [Color figure can be viewed in the online issue, which is available at www.annalsofneurology.org.]

**Figure 5:**
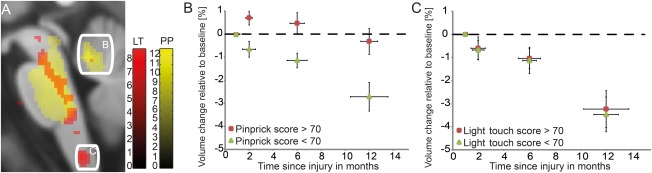
Correlation between progressive volumetric changes in the brain and sensory outcome. (A) Overlay of statistical parametric maps (uncorrected *p* < 0.001, for illustrative purposes) shows a correlation between cerebellum and pinprick (PP) score at 12 months (yellow, data of peak voxel plotted in B) and between the medulla oblongata and light touch (LT) score at 12 months (red, data of peak voxel plotted in C). The color bars indicate the corresponding *t* scores. (B) Greater volumetric decreases over time in patients with lower pinprick outcome at 12 months. (C) No difference in volumetric decreases over time in patients with lower light touch outcome at 12 months. Vertical error bars show standard error (SE) for volumetric change in selected area of interest, and horizontal error bars show SE for scan intervals. An arbitrary threshold for clinical outcome at 12 months was set to 70 (for illustrative purposes).

ISNCSCI pinprick scores at 12 months were associated with rate of cord area decrease (*p* = 0.020, *r* ^2^ = 0.76; Fig 4A). ISNCSCI pinprick scores at 12 months were associated with rate of volumetric changes in the right cerebellum (x: 2, y: −47, z: −18, *z* score = 5.80, *p* < 0.001, cluster extent = 197; x: 12, y: −62, z: −16, *z* score = 4.87, *p* = 0.017, cluster extent = 75; Fig 5A, B) and ISNCSCI light touch scores with rate of volumetric changes in the dorsal columns at the level of the medulla oblongata (x: 14, y: −29, z: −42, *z* score = 4.07, *p* = 0.023, cluster extent = 31; x: −8, y: 36, z: −48, *z* score = 3.88, *p* = 0.016, cluster extent = 76; x: 5, y: −45, z: −63, *z* score = 4.76, *p* = 0.026, cluster extent = 76, latter cluster only significant at peak voxel; see Fig 5A, C).

At 12 months, ISNCSCI pinprick scores were associated with spinal cord MT (*p* = 0.005, *r* ^2^ = 0.82; see Fig 4B).

## Discussion

This study shows progressive volumetric decreases along the sensory system from the earliest stage after SCI. The evaluation of myelin‐sensitive qMRI supports the hypothesis that the observed volumetric changes relate to changes in the underlying myeloarchitecture. From the clinical perspective, the observed structural changes in the cord and brain were related to the extent of sensory outcome but were not related to neuropathic pain. Thus, both the motor^9^ and sensory systems are susceptible to atrophy early after injury. Therefore, the MRI changes could be considered as complementing the clinical assessment for monitoring sensory impairment and outcome during the course of rehabilitation and treatment interventions following SCI.

### Evolution of Structural Changes from the Spinal Cord to the Brain

During the first year after traumatic SCI, the cord area declines by 7%,^9^ with a reduction of up to 30% 15 years postinjury.^12^, ^53^ Besides the overall reduction of cord area, morphometric changes may provide further insight into tract‐specific changes, as reductions of the APW have been associated with sensory impairment and the LRW with motor function in chronic SCI.^12^ Here, we assessed the progressive shape changes of the cord (ie, APW and LRW). In agreement with the observations in chronic SCI patients,^12^ shape deformations occurred in both the APW and LRW. The change in the APW might reflect anterograde degeneration occurring along the dorsal columns^12^, ^18^, ^19^ in parallel with retrograde degeneration of the descending tracts (eg, corticospinal tracts) captured by the LRW.^12^ The reductions of myelin‐sensitive MT and R1 parameters (at the same cord level) and of diffusion tensor metrics in the chronic injured spinal cord^11^, ^54^ may reflect ongoing changes of the myelin architecture that in turn contribute to the shape changes.

At the level of the brain, we found progressive volumetric decreases in areas involved in sensory processing (eg, brainstem, thalamus, ACC, S2, and insula) between baseline and 12 months. Alterations in structure and function in these areas^14^, ^18^, ^55^, ^56^ have been associated with impaired sensory processing and sensory discomfort in patients with SCI.^22^ Similar to findings in the spinal cord, myelin‐sensitive R1 and MT paralleled the volumetric changes observed in the thalamus, cerebellum, and medulla oblongata comprising the dorsal columns. The bilateral decrease of both volume and myelin‐sensitive MR parameters within the same cortical areas is suggestive of ongoing structural changes responding to the SCI that in the present SCI cohort caused equally severe impairments on either side of the body. These changes are likely to involve changes at the level of cellular metabolism, blood flow, and functional depression, inducing a state of hypoactivity and shrinkage of sensory neurons and their axons.^57^ In accordance with motor system atrophy during the first year after injury,^9^ the magnitude of atrophy within the sensory system was evident in both incomplete and complete paraplegic and tetraplegic patients. This finding is of interest, as patients with a very chronic SCI show level‐dependent spinal atrophy, with more pronounced atrophic changes in those patients with higher lesion levels.^12^ Thus, level‐dependent atrophy (ie, lesions in the cervical cord impact the structural integrity of a greater number of fibers and neurons than a comparable thoracic lesion) may only become distinguishable at later disease stages of SCI. However, we are aware that the sample size of our study is rather small and weak effects might not have been detected. To detect the full spectrum of potential changes, future multicenter studies with the advantage of collecting large sample sizes are required. Our results provide the necessary motivation and evidence for conducting such expensive studies.

### Clinical Associations with Structural Changes

Crucially, the rate of volume change and the reduction of myelin‐sensitive structural measures in the cord and volume changes in the brain related quantitatively to sensory deficits. In other words, faster atrophy and greater decreases in myelin‐sensitive markers were seen in ascending spinal pathways and sensory‐specific brain areas in patients with greater loss of pain (pinprick) and light touch (brush) sensation. At the cervical cord and the medulla oblongata level, long‐distance Wallerian degeneration of primary afferents within the spinothalamic tracts as wells as dorsal columns is a likely pathophysiological substrate underlying the clinicopathological associations of pinprick and light touch.^58^, ^59^ Within the brainstem and cerebellum (both receivers of afferent spinal inputs^18^, ^48^), the interpretation of the association between trauma‐induced atrophy rate and light touch and pinprick outcome is more complex. In addition to Wallerian degeneration of ascending fiber pathways that arise from the spinal cord, transsynaptic changes affecting the structure and function of sensory relay nuclei in the brainstem and neurons within the cerebellum (eg, Purkinje cells) occur as a consequence of deafferentation.^18^, ^21^, ^60^ The relationship between greater cerebellar atrophy rate and worse pinprick outcome is therefore interesting, as the cerebellum may also be involved in trauma‐induced maladaptive processing of afferent sensory inputs (eg, nociception)^61^ alongside its role as a comparator for errors in somatosensory processing^62^ resulting in motor impairment.^48^ Thus, a trauma‐dependent altered structure function relationship due to deafferentation could explain why patients with greater sensory dysfunction (ie, worse protopathic sensation) show more severe atrophy. The shape changes (APW, LRW) of the cord could not be associated with specific sensory outcomes. Therefore, the rather gross geometrical changes (APW, LRW) along the axis might only become responsive to sensory impairment in the very chronic phase of injury.^12^

The clinical standard to determine the degree of sensory disability—that is, epicritic and protopathic sensation—after spinal cord damage is the testing of light touch and pinprick sensation, respectively. Somatosensory^63^ and contact heat evoked potentials^64^ have been shown to provide complementary insights into the pathophysiology underlying sensory deficits, as they reveal subclinical afferent sparing beyond pinprick and light touch sensation. Thus, future serial studies will integrate these complementary measures as they have the potential to reveal pathology to anatomically distinct pathways with greater resolution (ie, dorsal columns and spinothalamic tract).^65^

Although neuropathic pain developed in this patient group over time, neither the onset nor the intensity of neuropathic pain was linked to neurodegeneration (eg, reduction in volume decline and myelin) during the first year of injury. Whereas loss of sensory input is generally induced by neural damage and disconnection within ascending sensory pathways,^18^, ^19^ most important mechanisms underlying neuropathic pain in the subacute phase of injury originate in the brain with complex interactions of the spinal and supraspinal neuronal circuits.^15^, ^66^, ^67^ Studies focusing on the chronic phase were able to associate structural changes in brain regions with nociceptive processing (ie, thalamus, prefrontal cortex, insular cortex, amygdala, and premotor cortex), although the ultimate mechanisms remain unclear.^67^ Future multimodal studies integrating serial structural and functional MRI and electrophysiological assessments of pain are needed to address this issue further.

### Limitations

Our study had some limitations. First, controls were on average 12 years younger than patients. We included age as a covariate in all statistical analyses to exclude any (linear) age‐related effects, although the relationship between age and cord area is not significant.^52^ Moreover, our analysis revealed that the brain volume trajectories were not significantly associated with age nor did the adjustment of age influence the latter significantly. However, the validity of the adjustment can only be asserted confidently for patients and controls younger than 55 years, because there were no controls older than 55 years. Second, although computational morphometry can reveal disease‐specific changes over time, it is not biologically specific. In this study, these morphometric changes were paralleled by changes in qMRI metrics that are sensitive to underlying tissue microstructure. Measures of MT provide information about the macromolecular content of the microstructural environment. Although not a direct measure of myelin, postmortem validation has shown high correspondence between MT‐based measures and myelin staining,^68^, ^69^ indicating that myelin is a significant contributor to this measure. Even though there are multiple contributors to the measured R1, including water and iron content, the contribution from macromolecular components has been shown to dominate.^31^, ^70^ Therefore, we interpret the changes observed in this study as being consistent with axonal demyelination that contributes to volumetric changes within the sensory system. Third, defining compartments of the spinal cord to distinguish ascending and descending pathways is not completely accurate, as tracts located laterally also transmit ascending information (eg, spinocerebellar and spinothalamic tracts). Finally, the mean intervals between the MRIs in patients during follow‐up varied, but importantly as a result the dropout rate was minimal (compliance of 95%). We note that we accounted for this variability in the linear trajectory analyses of cord and brain MRI parameters by explicitly modeling the timing of the MRI acquisitions for all subjects.

### Conclusion

The neuroimaging biomarkers applied for the first time were sensitive to dynamic volumetric changes of the sensory system at both the spinal cord and supraspinal level that are likely to be associated with changes in myelin architecture. Importantly, slower rates of atrophy were associated with less severe sensory disturbance. These findings indicate that not only the motor but also the sensory system undergoes remote (spinal and supraspinal) changes that complement clinical measures of recovery, although the underlying pathophysiological mechanisms are yet to be elucidated.

## Authorship

The study was designed by P.F., N.W., A.J.T., and A.C. The data were analyzed by P.G., M.F.C., P.F., N.W., and J.A. All authors wrote and reviewed the paper.

## Potential Conflicts of Interest

N.W.: grants, European Union, MRC, UCL SLMS, Wings for Life; institutional research agreement, Siemens; consultancy, UCL Consultants; patent, DE 10319037 A1 (licensee, University of Tübingen). A.J.T.: consultancy, Biogen Idec, Novartis, Medday, Eisai, Genzyme; speaking fees, Novartis, Teva, EXCEMED, Remedica; honorarium, Sage Publications (as Editor in Chief, *Multiple Sclerosis*). P.F.: grant, Wings for Life.

## References

[1] Zariffa J , Kramer JLK , Fawcett JW , et al. Characterization of neurological recovery following traumatic sensorimotor complete thoracic spinal cord injury. Spinal Cord 2011;49:463–471. 20938451 10.1038/sc.2010.140

[2] Furlan JC , Noonan V , Singh A , Fehlings MG. Assessment of impairment in patients with acute traumatic spinal cord injury: a systematic review of the literature. J Neurotrauma 2011;28:1445–1477. 20030559 10.1089/neu.2009.1152 PMC3143408

[3] Siddall PJ , Loeser JD. Pain following spinal cord injury. Spinal Cord 2001;39:63–73. 11402361 10.1038/sj.sc.3101116

[4] Siddall PJ , McClelland JM , Rutkowski SB , Cousins MJ. A longitudinal study of the prevalence and characteristics of pain in the first 5 years following spinal cord injury. Pain 2003;103:249–257. 12791431 10.1016/S0304-3959(02)00452-9

[5] Buss A , Schwab ME. Sequential loss of myelin proteins during Wallerian degeneration in the rat spinal cord. Glia 2003;42:424–432. 12730963 10.1002/glia.10220

[6] Jones EG , Pons TP. Thalamic and brainstem contributions to large‐scale plasticity of primate somatosensory cortex. Science 1998;282:1121–1125. 9804550 10.1126/science.282.5391.1121

[7] Ghosh A , Sydekum E , Haiss F , et al. Functional and anatomical reorganization of the sensory‐motor cortex after incomplete spinal cord injury in adult rats. J Neurosci 2009;29:12210–12219. 19793979 10.1523/JNEUROSCI.1828-09.2009 PMC6666156

[8] Yague JG , Foffani G , Aguilar J. Cortical hyperexcitability in response to preserved spinothalamic inputs immediately after spinal cord hemisection. Exp Neurol 2011;227:252–263. 21093438 10.1016/j.expneurol.2010.11.011

[9] Freund P , Weiskopf N , Ashburner J , et al. MRI investigation of the sensorimotor cortex and the corticospinal tract after acute spinal cord injury: a prospective longitudinal study. Lancet Neurol 2013;12:873–881. 23827394 10.1016/S1474-4422(13)70146-7 PMC3744750

[10] Miyanji F , Furlan JC , Aarabi B , et al. Acute cervical traumatic spinal cord injury: MR imaging findings correlated with neurologic outcome—prospective study with 100 consecutive patients. Radiology 2007;243:820–827. 17431129 10.1148/radiol.2433060583

[11] Cohen‐Adad J , El Mendili M‐M , Lehéricy S , et al. Demyelination and degeneration in the injured human spinal cord detected with diffusion and magnetization transfer MRI. Neuroimage 2011;55:1024–1033. 21232610 10.1016/j.neuroimage.2010.11.089

[12] Lundell H , Barthelemy D , Skimminge A , et al. Independent spinal cord atrophy measures correlate to motor and sensory deficits in individuals with spinal cord injury. Spinal Cord 2011;49:70–75. 20697420 10.1038/sc.2010.87

[13] Wrigley PJ , Gustin SM , Macey PM , et al. Anatomical changes in human motor cortex and motor pathways following complete thoracic spinal cord injury. Cereb Cortex 2009;19:224–232. 18483004 10.1093/cercor/bhn072

[14] Gustin SM , Wrigley PJ , Siddall PJ , Henderson LA. Brain anatomy changes associated with persistent neuropathic pain following spinal cord injury. Cereb Cortex 2010;20:1409–1419. 19815621 10.1093/cercor/bhp205

[15] Mole TB , MacIver K , Sluming V , et al. Specific brain morphometric changes in spinal cord injury with and without neuropathic pain. Neuroimage Clin 2014;5:28–35. 24936434 10.1016/j.nicl.2014.05.014 PMC4055864

[16] Cadotte DW , Bosma R , Mikulis D , et al. Plasticity of the injured human spinal cord: insights revealed by spinal cord functional MRI. PLoS One 2012;7:e45560. 23029097 10.1371/journal.pone.0045560 PMC3446947

[17] Stroman PW , Tomanek B , Krause V , et al. Mapping of neuronal function in the healthy and injured human spinal cord with spinal fMRI. Neuroimage 2002;17:1854–1860. 12498759 10.1006/nimg.2002.1305

[18] Jain N , Florence SL , Qi H , Kaas JH. Growth of new brainstem connections in adult monkeys with massive sensory loss. Proc Natl Acad Sci U S A 2000;97:5546–5550. 10779564 10.1073/pnas.090572597 PMC25865

[19] Chen LM , Qi H‐X , Kaas JH. Dynamic reorganization of digit representations in somatosensory cortex of nonhuman primates after spinal cord injury. J Neurosci 2012;32:14649–14663. 23077051 10.1523/JNEUROSCI.1841-12.2012 PMC3498942

[20] Aguilar J , Humanes‐Valera D , Alonso‐Calviño E , et al. Spinal cord injury immediately changes the state of the brain. J Neurosci 2010;30:7528–7537. 20519527 10.1523/JNEUROSCI.0379-10.2010 PMC3842476

[21] Villiger M , Grabher P , Kiper D , Freund P. Relationship between structural brainstem and brain plasticity and lower‐limb training in spinal cord injury: a longitudinal pilot study. Front Hum Neurosci 2015;9:1–10. 25999842 10.3389/fnhum.2015.00254 PMC4420931

[22] Garcia‐Larrea L , Peyron R. Pain matrices and neuropathic pain matrices: a review. Pain 2013;154:S29–S43. 24021862 10.1016/j.pain.2013.09.001

[23] Weiskopf N , Mohammadi S , Lutti A , Callaghan MF. Advances in MRI‐based computational neuroanatomy: from morphometry to in‐vivo histology. Curr Opin Neurol 2015;28:313–322. 26132532 10.1097/WCO.0000000000000222

[24] Huber E , Curt A , Freund P. Tracking trauma‐induced structural and functional changes above the level of spinal cord injury. Curr Opin Neurol 2015;28:365–372. 26110798 10.1097/WCO.0000000000000224

[25] Ashburner J , Ridgway GR. Symmetric diffeomorphic modeling of longitudinal structural MRI. Front Neurosci 2013;6:1–19. 10.3389/fnins.2012.00197 PMC3564017 23386806

[26] Weiskopf N , Lutti A , Helms G , et al. Unified segmentation based correction of R1 brain maps for RF transmit field inhomogeneities (UNICORT). Neuroimage 2011;54:2116–2124. 20965260 10.1016/j.neuroimage.2010.10.023 PMC3018573

[27] Kirshblum SC , Waring W , Biering‐Sorensen F , et al. Reference for the 2011 revision of the International Standards for Neurological Classification of Spinal Cord Injury. J Spinal Cord Med 2011;34:547–554. 22330109 10.1179/107902611X13186000420242 PMC3232637

[28] Anderson K , Aito S , Atkins M , et al. Functional recovery measures for spinal cord injury: an evidence‐based review for clinical practice and research. J Spinal Cord Med 2008;31:133–144. 18581660 10.1080/10790268.2008.11760704 PMC2578796

[29] Widerström‐Noga E , Biering‐Sørensen F , Bryce TN , et al. The International Spinal Cord Injury Pain Basic Data Set (version 2.0). Spinal Cord 2014;52:282–286. 24469147 10.1038/sc.2014.4

[30] Hassanpour K , Hotz‐Boendermaker S , Dokladal P , Curt A. Low depressive symptoms in acute spinal cord injury compared to other neurological disorders. J Neurol 2012;259:1142–1150. 22095042 10.1007/s00415-011-6316-2

[31] Rooney WD , Johnson G , Li X , et al. Magnetic field and tissue dependencies of human brain longitudinal 1H2O relaxation in vivo. Magn Reson Med 2007;57:308–318. 17260370 10.1002/mrm.21122

[32] Helms G , Dathe H , Kallenberg K , Dechent P. High‐resolution maps of magnetization transfer with inherent correction for RF inhomogeneity and T1 relaxation obtained from 3D FLASH MRI. Magn Reson Med 2008;60:1396–1407. 19025906 10.1002/mrm.21732

[33] Weiskopf N , Suckling J , Williams G , et al. Quantitative multi‐parameter mapping of R1, PD(*), MT, and R2(*) at 3T: a multi‐center validation. Front Neurosci 2013;7:95. 23772204 10.3389/fnins.2013.00095 PMC3677134

[34] Tardif CL , Collins DL , Pike GB. Sensitivity of voxel‐based morphometry analysis to choice of imaging protocol at 3 T. Neuroimage 2009;44:827–838. 18996205 10.1016/j.neuroimage.2008.09.053

[35] Horsfield MA , Sala S , Neema M , et al. Rapid semi‐automatic segmentation of the spinal cord from magnetic resonance images: application in multiple sclerosis. Neuroimage 2010;50:446–455. 20060481 10.1016/j.neuroimage.2009.12.121 PMC2830007

[36] Ashburner J , Friston KJ. Unified segmentation. Neuroimage 2005;26:839–851. 15955494 10.1016/j.neuroimage.2005.02.018

[37] Ashburner J. A fast diffeomorphic image registration algorithm. Neuroimage 2007;38:95–113. 17761438 10.1016/j.neuroimage.2007.07.007

[38] Draganski B , Ashburner J , Hutton C , et al. Regional specificity of MRI contrast parameter changes in normal ageing revealed by voxel‐based quantification (VBQ). Neuroimage 2011;55:1423–1434. 21277375 10.1016/j.neuroimage.2011.01.052 PMC3093621

[39] Helms G , Draganski B , Frackowiak R , et al. Improved segmentation of deep brain grey matter structures using magnetization transfer (MT) parameter maps. Neuroimage 2009;47:194–198. 19344771 10.1016/j.neuroimage.2009.03.053 PMC2694257

[40] Harkins KD , Xu J , Dula AN , et al. The microstructural correlates of t1 in white matter. Magn Reson Med 2015;00. doi: 10.1002/mrm.25709. 10.1002/mrm.25709 PMC4624612 25920491

[41] Murray EA , Mishkin M. Relative contributions of SII and area 5 to tactile discrimination in monkeys. Behav Brain Res 1984;11:67–83. 6696789 10.1016/0166-4328(84)90009-3

[42] Ogino Y , Nemoto H , Inui K , et al. Inner experience of pain: imagination of pain while viewing images showing painful events forms subjective pain representation in human brain. Cereb Cortex 2007;17:1139–1146. 16855007 10.1093/cercor/bhl023

[43] Ray S , Hsiao SS , Crone NE , et al. Effect of stimulus intensity on the spike‐local field potential relationship in the secondary somatosensory cortex. J Neurosci 2008;28:7334–7343. 18632937 10.1523/JNEUROSCI.1588-08.2008 PMC2597587

[44] Eippert F , Bingel U , Schoell ED , et al. Activation of the opioidergic descending pain control system underlies placebo analgesia. Neuron 2009;63:533–543. 19709634 10.1016/j.neuron.2009.07.014

[45] Eickhoff SB , Stephan KE , Mohlberg H , et al. A new SPM toolbox for combining probabilistic cytoarchitectonic maps and functional imaging data. Neuroimage 2005;25:1325–1335. 15850749 10.1016/j.neuroimage.2004.12.034

[46] Diedrichsen J. A spatially unbiased atlas template of the human cerebellum. Neuroimage 2006;33:127–138. 16904911 10.1016/j.neuroimage.2006.05.056

[47] Jahn K , Deutschländer A , Stephan T , et al. Imaging human supraspinal locomotor centers in brainstem and cerebellum. Neuroimage 2008;39:786–792. 18029199 10.1016/j.neuroimage.2007.09.047

[48] Moulton EA , Schmahmann JD , Becerra L , Borsook D. The cerebellum and pain: passive integrator or active participator? Brain Res Rev 2010;65:14–27. 20553761 10.1016/j.brainresrev.2010.05.005 PMC2943015

[49] Benarroch EE. Pedunculopontine nucleus: functional organization and clinical implications. Neurology 2013;80:1148–1155. 23509047 10.1212/WNL.0b013e3182886a76

[50] Tattersall TL , Stratton PG , Coyne TJ , et al. Imagined gait modulates neuronal network dynamics in the human pedunculopontine nucleus. Nat Neurosci 2014;17:449–454. 24487235 10.1038/nn.3642

[51] Friston KJ , Worsley KJ , Frackowiak RS , et al. Assessing the significance of focal activations using their spatial extent. Hum Brain Mapp 1994;1:210–220. 24578041 10.1002/hbm.460010306

[52] Callaghan MF , Freund P , Draganski B , et al. Widespread age‐related differences in the human brain microstructure revealed by quantitative magnetic resonance imaging. Neurobiol Aging 2014;35:1862–1872. 24656835 10.1016/j.neurobiolaging.2014.02.008 PMC4024196

[53] Freund P , Weiskopf N , Ward NS , et al. Disability, atrophy and cortical reorganization following spinal cord injury. Brain 2011;134(pt 6):1610–1622. 21586596 10.1093/brain/awr093 PMC3102242

[54] Freund P , Schneider T , Nagy Z , et al. Degeneration of the injured cervical cord is associated with remote changes in corticospinal tract integrity and upper limb impairment. PLoS One 2012;7:e51729. 23251612 10.1371/journal.pone.0051729 PMC3520920

[55] Gustin SM , Wrigley PJ , Youssef AM , et al. Thalamic activity and biochemical changes in individuals with neuropathic pain after spinal cord injury. Pain 2014;155:1027–1036. 24530612 10.1016/j.pain.2014.02.008 PMC4410007

[56] Widerström‐Noga E , Pattany PM , Cruz‐Almeida Y , et al. Metabolite concentrations in the anterior cingulate cortex predict high neuropathic pain impact after spinal cord injury. Pain 2013;154:204–212. 23141478 10.1016/j.pain.2012.07.022 PMC3670594

[57] Moxon KA , Oliviero A , Aguilar J , Foffani G. Cortical reorganization after spinal cord injury: Always for good? Neuroscience 2014;283:78–94. 24997269 10.1016/j.neuroscience.2014.06.056 PMC4556279

[58] Zhang J , Jones M , DeBoy CA , et al. Diffusion tensor magnetic resonance imaging of Wallerian degeneration in rat spinal cord after dorsal root axotomy. J Neurosci 2009;29:3160–3171. 19279253 10.1523/JNEUROSCI.3941-08.2009 PMC2683764

[59] Kaas JH , Qi H‐X , Burish MJ , et al. Cortical and subcortical plasticity in the brains of humans, primates, and rats after damage to sensory afferents in the dorsal columns of the spinal cord. Exp Neurol 2008;209:407–416. 17692844 10.1016/j.expneurol.2007.06.014 PMC2268113

[60] Kaas JH , Florence SL , Jain N. Subcortical contributions to massive cortical reorganizations. Neuron 1999;22:657–660. 10230786 10.1016/s0896-6273(00)80725-4

[61] Cerminara NL , Koutsikou S , Lumb BM , Apps R. The periaqueductal grey modulates sensory input to the cerebellum: a role in coping behaviour? Eur J Neurosci 2009;29:2197–2206. 19453624 10.1111/j.1460-9568.2009.06760.x

[62] Apps R , Garwicz M. Anatomical and physiological foundations of cerebellar information processing. Nat Rev Neurosci 2005;6:297–311. 15803161 10.1038/nrn1646

[63] Kuhn F , Halder P , Spiess MR , Schubert M. One‐year evolution of ulnar somatosensory potentials after trauma in 365 tetraplegic patients: early prediction of potential upper limb function. J Neurotrauma 2012;29:1829–1837. 22519951 10.1089/neu.2011.2277

[64] Haefeli J , Kramer JLK , Blum J , Curt A. Assessment of spinothalamic tract function beyond pinprick in spinal cord lesions: a contact heat evoked potential study. Neurorehabil Neural Repair 2013;28:494–503. 24379083 10.1177/1545968313517755

[65] Ellaway PH , Kuppuswamy A , Balasubramaniam AV , et al. Development of quantitative and sensitive assessments of physiological and functional outcome during recovery from spinal cord injury: a clinical initiative. Brain Res Bull 2011;84:343–357. 20728509 10.1016/j.brainresbull.2010.08.007

[66] Makin TR , Scholz J , Filippini N , et al. Phantom pain is associated with preserved structure and function in the former hand area. Nat Commun 2013;4:1570. 23463013 10.1038/ncomms2571 PMC3615341

[67] Wrigley PJ , Press SR , Gustin SM , et al. Neuropathic pain and primary somatosensory cortex reorganization following spinal cord injury. Pain 2009;141:52–59. 19027233 10.1016/j.pain.2008.10.007

[68] Schmierer K , Scaravilli F , Altmann DR , et al. Magnetization transfer ratio and myelin in postmortem multiple sclerosis brain. Ann Neurol 2004;56:407–415. 15349868 10.1002/ana.20202

[69] Turati L , Moscatelli M , Mastropietro A , et al. In vivo quantitative magnetization transfer imaging correlates with histology during de‐ and remyelination in cuprizone‐treated mice. NMR Biomed 2015;28:327–337. 25639498 10.1002/nbm.3253

[70] Callaghan MF , Helms G , Lutti A , et al. A general linear relaxometry model of R1 using imaging data. Magn Reson Med 2015;73:1309–1314. 24700606 10.1002/mrm.25210 PMC4359013

